# Biomechanical characterisation of the human nasal cartilages; implications for tissue engineering

**DOI:** 10.1007/s10856-015-5619-8

**Published:** 2015-12-16

**Authors:** M. F. Griffin, Y. Premakumar, A. M. Seifalian, M. Szarko, P. E. M. Butler

**Affiliations:** Centre for Nanotechnology & Regenerative Medicine, UCL Division of Surgery & Interventional Science, University College London, London, UK; Anatomical Sciences, Institute for Medical and Biomedical Education, St. George’s, University of London, London, UK; Department of Plastic and Reconstructive Surgery, Royal Free Hampstead NHS Trust Hospital, London, UK

## Abstract

**Abstract:**

Nasal reconstruction is currently performed using autologous grafts provides but is limited by donor site morbidity, tissue availability and potentially graft failure. Additionally, current alternative alloplastic materials are limited by their high extrusion and infection rates. Matching mechanical properties of synthetic materials to the native tissue they are replacing has shown to be important in the biocompatibility of implants. To date the mechanical properties of the human nasal cartilages has not been studied in depth to be able to create tissue-engineered replacements with similar mechanical properties to native tissue. The young’s modulus was characterized in compression on fresh-frozen human cadaveric septal, alar, and lateral cartilage. Due to the functional differences experienced by the various aspects of the septal cartilage, 16 regions were evaluated with an average elastic modulus of 2.72 ± 0.63 MPa. Furthermore, the posterior septum was found to be significantly stiffer than the anterior septum (*p* < 0.01). The medial and lateral alar cartilages were tested at four points with an elastic modulus ranging from 2.09 ± 0.81 MPa, with no significant difference between the cartilages (*p* < 0.78). The lateral cartilage was tested once in all cadavers with an average elastic modulus of 0.98 ± 0.29 MPa. In conclusion, this study provides new information on the compressive mechanical properties of the human nasal cartilage, allowing surgeons to have a better understanding of the difference between the mechanical properties of the individual nasal cartilages. This study has provided a reference, by which tissue-engineered should be developed for effective cartilage replacements for nasal reconstruction.

**Graphical Abstract:**

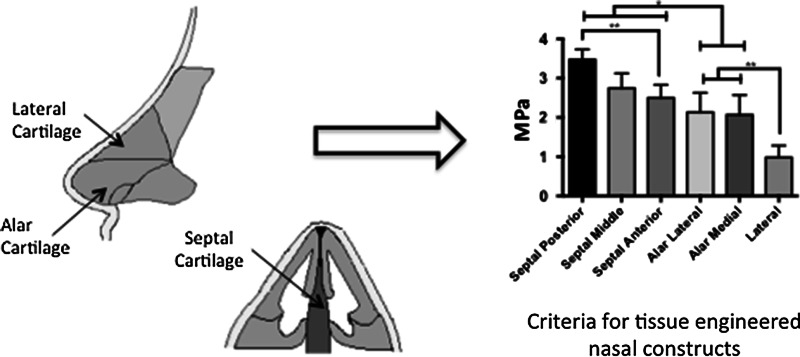

**Electronic supplementary material:**

The online version of this article (doi:10.1007/s10856-015-5619-8) contains supplementary material, which is available to authorized users.

## Introduction

Nasal defects are caused by several pathologies including trauma, cancer, dermatological disease and congenital malformations [1]. This devastating facial disfigurement causes physical and psychological difficulties for patients affecting their social life, interpersonal relationships and ability to work. The primary aim of nasal reconstruction is to create a patent airway passage for the patient, achieve wound healing and an inconspicuous nasal organ to improve their self-esteem and quality of life. To achieve this and replace the cartilage framework of the nose, patients own tissue is primarily used, including the auricular and costal cartilage [1]. However, despite being regarded as the optimal restorative technique, this causes significant donor site morbidity and limited reconstructive options due to the minimal availability of the cartilage. This has caused surgeons to utilise synthetic materials to restore nasal organs [1]. Several materials have been introduced for nasal reconstruction. Silicone, has been used for several years but the non-porous characteristics prevents integration with the host tissue. Porous, high density polyethylene, Medpor is the currently used porous biocompatible synthetic material to restore the facial skeleton. Unfortunately, high levels of infection and extrusion limits currently available materials [1]. Therefore, there is an unmet clinical need to create a suitable material for nasal reconstruction to provide better outcomes for patients [1].

With the ever-increasing advances in tissue engineering, it is likely that tissue-engineered cartilage replacements will overcome autologous cartilage as the traditional material for nasal reconstruction. Mechanical properties are important to determine a materials ability to withstand compression, tensile and shear stress forces. To date, the mechanical properties of the human nasal cartilage has not been clearly defined which limits the ability to create a nasal construct with similar properties as the native tissue [2]. Few studies, have tried to characterize the mechanical properties of animal septum [2–4] with only a few studies to reporting mechanical properties of the human septum [2, 6, 7].

With the complex geometry of the nasal cartilages the present study aimed to identify the mechanical properties of all the human nasal cartilages and to compare them to one another. The aims of this study were to create a (a) mechanical map of the nasal cartilages in compression using human cadaver specimens and support these findings with a (b) histological map and in so doing provide a detailed understanding of both the structure and function of the nasal cartilages.

## Materials and methods

### Cartilage harvest

Fresh-Frozen human nasal cartilage was harvested from the nasal structures of 15 male cadaveric specimens (average age 56 ± 15 years). Following harvest the nasal construct was placed into sterile normal saline at 37.5 °C to defrost the nasal cartilages for further dissection. Firstly, the skin and fascia was dissected from the cartilaginous framework. Following this procedure, the cartilage specimens were dissected into the three individual nasal cartilages for compressive mechanical testing.

### Mechanical testing

**Fig. 1:**
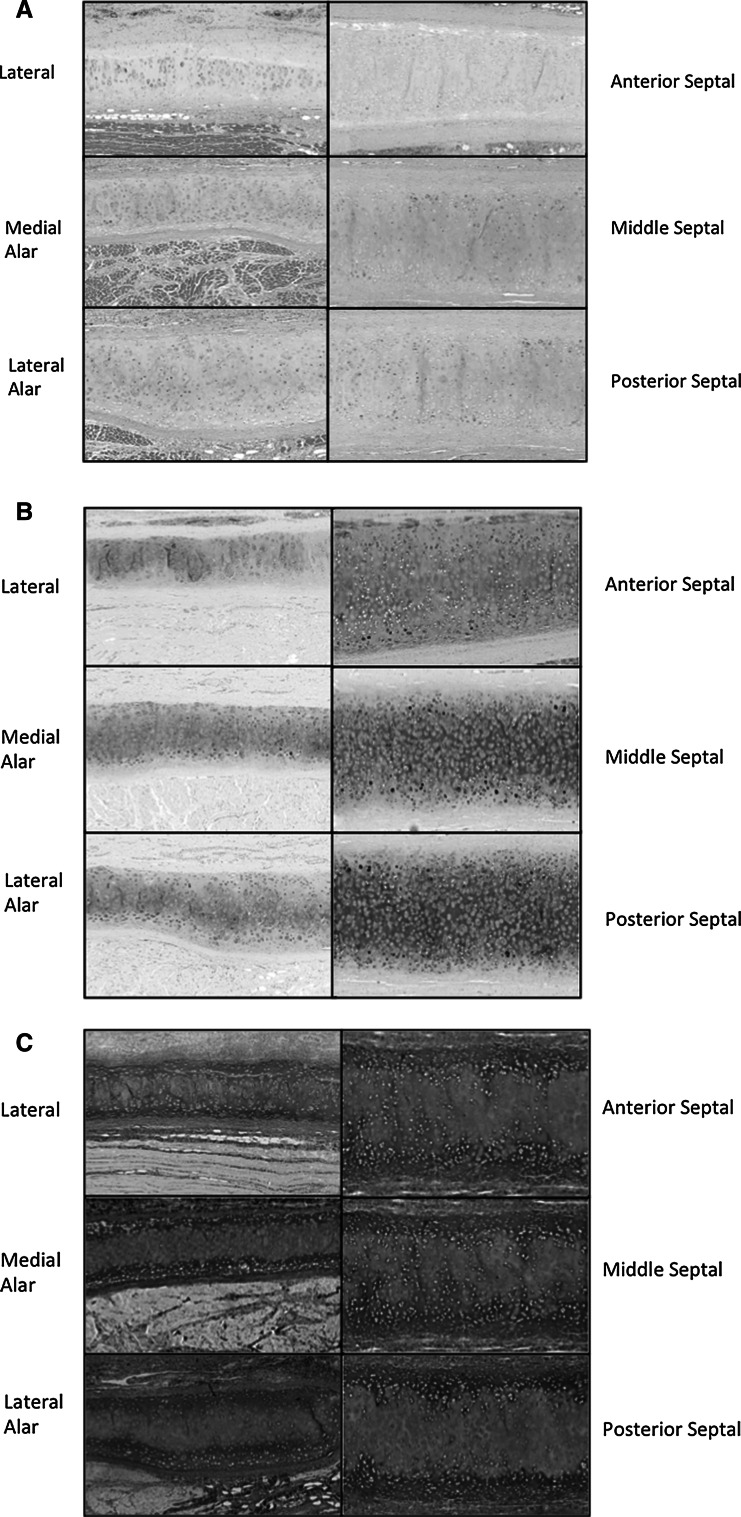
Histological analysis of the nasal cartilages using **a** H&E stain for structure, **b** Alcian blue and PAS stain for glycoprotein content and **c** Elastin Van Gieson stain for elastin and collagen (EVG)

After removal of the skin and fascia, the nasal cartilages were tested as illustrated in supplementary Fig. 1. The thicknesses of the nasal cartilages were measured using a digital vernier calipers (supplementary Fig. 2). Once the nose was fully denuded the following guidelines were utilized referring to Fig. 1 to cut the nose into the 26 areas. The 26 points were initially chosen to provide the most detailed mechanical and histological map of the nasal cartilage, which covered all the anatomical structures of the nasal framework including the septal, alar and lateral cartilages. Cartilage samples were compressed using indentation using a Mach-1 materials testing machine in a hydrated environment at room temperature (Biomomentum, Canada). Each sample was loaded to 300 g at 1 mm/s via the 1 kg load cell. After the 300 g was reached, the tissue was allowed to relax for 15 min (a time point sufficient to control for stress equilibrium). The size of the indentor was 0.2 mm. Using an indentor much smaller than radius than the sample diameter eliminated edge effect. This indentor was chosen for all samples with approximately 8 times greater diameter than the indenter of the cartilage, the cartilage under load will react essentially as if it were part of an indefinite sample [8, 9]. The resulting young’s modulus and stress relaxation properties calculations were calculated as previously described [8, 9]. In addition to Young’s elastic modulus, the stress-time slope was used to measure the stiffness of the anatomical ultrastructure (i.e. removal of strain which normalizes thickness to displacement).

#### Cartilage preparation

##### Lateral cartilage

Using electronic calipers the lateral cartilage the center of each lateral cartilage was found by measuring the length and width of each lateral cartilage. The lateral cartilage was then orientated flat for compressive testing with the outer side facing upwards (supplementary Fig. 1a). Both the left and right lateral cartilage was tested using this protocol.

##### Alar cartilage

Using electronic calipers the medial and lateral alar cartilages were divided into two by calculating their length and width measurement. The centre of each of the halves of the alar cartilage was calculated, prior to the outer side being placed completely flat for compressive testing (supplementary Fig. 1b).

##### Septal cartilage

Using electronic calipers the septal cartilages were divided into four sections from anterior and posterior calculated by their length measurement. Posterior was classified as the end of the cartilage that was attached to the facial bones. The four sections were then sub-sectioned from top to bottom as per their width. The samples were then laid completely flat with the right side of the septum always being used for compressive testing (supplementary Fig. 1c).

### Histological testing

Tissue was formalin-fixed, paraffin-embedded and sectioned at 4 H&E, Alcian blue & PAS staining and Elastin Van Gieson (EVG) stains were conducted according to standard protocols, then photographed with a slide scanner (Nanozoom Slide Scanner) at ×10 magnification.

### Data analysis

Comparisons between the nasal cartilages were analysed statistically using one-way analysis of variance (ANOVA) with Tukey HSD post hoc analysis (JMP, v10; North Carolina, USA). Significance was described as *p* < 0.05. Kaleida-graph (v.4.1, Pennsylvania, USA) was used for graphically representing data.

## Results

### Mechanical testing of the nasal cartilages

After calculating and comparing the thickness of the different nasal cartilages, it was observed that the septum (posterior 2.32 ± 0.22 mm, middle 2.19 ± 0.22 mm and anterior 1.97 ± 0.12 mm) was significantly thicker than the alar cartilages (lateral 1.40 ± 0.24 mm and medial 1.54 ± 0.28 mm, *p* < 0.05), which was thicker than the lateral cartilages (0.76 ± 0.11 mm, *p* < 0.05) (supplementary Fig. 2).

A young’s elastic modulus was observed for each of the nasal cartilages in compression. The nasal cartilages had different compressive properties, septal 2.72 ± 0.63 MPa, alar 2.09 ± 0.81 MPa and lateral 0.98 ± 0.29 MPa (Table 1, supplementary Fig. 3). Overall the septal cartilage was significantly stiffer than the alar, which was significantly stiffer and the lateral cartilages in compression (*p* < 0.05) (Table 1). The septal cartilage was observed to be significantly stiffer posteriorly than anteriorly (posterior septal 3.47 ± 0.26 MPa, middle septal 2.74 ± 0.37 MPa and anterior septal 2.50 ± 0.32 MPa, *p* < 0.05) but there was no difference superiorly to inferiorly (*p* < 0.76) (Table 1, supplementary Fig. 3). There was no difference in the stiffness between the medial and lateral alar cartilages (lateral 2.12 ± 0.5 MPa and medial 2.06 ± 0.5 MPa, *p* < 0.78) (Table 1, supplementary Fig. 3).

**Table 1:** Compressive and viscoelastic properties of the nasal cartilages based on anatomical structure of the nasal cartilages (MPa)

Mechanical property	Type of nasal cartilage (average, standard deviation)					
	Septal posterior	Septal middle	Septal anterior	Alar lateral	Alar medial	Lateral
Compression Young’s elastic modulus (MPa)	3.47 ± 0.26	2.74 ± 0.37	2.50 ± 0.32	2.12 ± 0.50	2.06 ± 0.50	0.98 ± 0.29
Final stress relaxation rate (MPa 10^−5^)	1.6 ± 0.40	1.33 ± 0.09	1.63 ± 0.42	3.4 ± 0.13	3.26 ± 0.15	1.46 ± 0.06
Final absolute relaxation rate (MPa)	0.23 ± 0.04	0.23 ± 0.05	0.21 ± 0.03	0.42 ± 0.06	0.41 ± 0.04	0.22 ± 0.03

* *P* < 0.05, ** *P* < 0.01, *** *P* < 0.001

To understand the complex geometry of the various anatomical structures within the nose, the strain (which normalises thickness to displacement) was removed. The resultant stiffness (slope of stress over time) is an indicative of the anatomical structure (i.e. alar arch). The mechanical stiffness irrespective of the thickness showed that the alar cartilage (lateral 0.62 ± 0.12 MPa and medial 0.64 ± 0.12 MPa) was significantly stiffer than the septal posterior (0.34 ± 0.15 MPa, middle 0.37 ± 0.13 MPa and anterior 0.24 MPa ± 0.12) and lateral cartilages (0.36 ± 0.19 MPa) (*p* < 0.05) (supplementary Fig. 4).

The final stress relaxation rate showed differences between the cartilages with the alar cartilage demonstrating a higher relaxation rate compared to the septal and lateral cartilages over 15 min (posterior septal 1.6 × 10^−5^ ± 0.40 MPa/s, middle septal 1.33 × 10^−5^ ± 0.09 MPa/s, anterior septal 1.63 × 10^−5^ ± 0.42 MPa/s, alar lateral 3.4 × 10^−5^ ± 0.13 MPa/s, alar medial 3.26 × 10^−5^ ± 0.15 MPa/s and lateral 1.46 × 10^−5^ ± 0.06 MPa/s, *p* < 0.001) (Table 1). The final absolute relaxation also showed the alar had a higher absolute relaxation rate than the septal and lateral cartilages (posterior septal 0.23 ± 0.04 MPa, middle septal 0.23 ± 0.05 MPa, anterior septal 0.21 ± 0.03 MPa, alar lateral 0.42 ± 0.06 MPa, alar medial 0.41 ± 0.04 MPa and lateral 0.22 ± 0.03 MPa, *p* < 0.001) (Table 1).

### Histological mapping

In order to understand the structural basis for the biomechanical differences between the nasal cartilages, the tissue was analysed by light microscopy (Fig. 1a–c). Using H&E, the structure of the nasal cartilages was investigated. All nasal cartilages demonstrated normal hyaline cartilage characteristics consisting of chondrocytes immersed within a homogenous cartilage. The chondrocytes were evenly distributed throughout the matrix in the alar and lateral nasal cartilages, but were more tightly packed in the septal cartilage. Alcian blue stained the acidic polysaccharides such as glycosaminoglycans in cartilages and PAS stained the proteoglycans of the nasal cartilages. The septal cartilage illustrated a greater degree of staining of the PAS and alcian blue. EVG staining determines the elastin (black) and collagen (red) content of the nasal cartilages. The cartilages showed no elastin only collagen staining. The alar cartilages did illustrate greater elastin staining in the surrounding subcutaneous tissue, likely reflecting the fact that they are the most mobile of the nasal structures and must be able to return to their original shape when deformed.

## Discussion

In this study, we have compared the mechanical properties of the human nasal cartilages. Due to the growing interest in tissue-engineered cartilage, the elastic modulus of native human nasal cartilage has become of great research interest. We have formulated a method by which to assess the nasal cartilages to allow a comparison between the different cartilages.

The human nasal cartilage showed different young’s elastic modulus in compression with the septum being significantly stiffer than the alar and lateral cartilages (*p* < 0.05). Richmon et al. tested the compressive properties of the human nasal septal cartilages observing the compressive properties to be in the range of 0.44–0.71 MPa, depending on orientation of the specimens [7]. In addition, Richmon et al. tested the human nasal septal cartilages in tension observing equilibrium modulus 3.01 ± 0.39 MPa, dynamic modulus 4.99 ± 0.49 MPa, strength 1.90 ± 0.24 MPa, and failure strain 0.35 ± 0.03 mm/mm [6]. Similarly, Westreich et al. tested the septal, lower and upper lateral cartilages under tension of five patients [2]. The stiffness of the cartilages showed considerable variability; lower lateral 1.82–15.28 MPa, upper lateral 5.43–28.63 MPa and septal 4.82–32.76 MPa. It is clear that is difficult to compare the results of different studies in terms of mechanical properties of the nasal cartilages. Lastly, Alkhan et al. observed the septal cartilage of 18 fresh cadavers to have an average elastic modulus of 1.39 MPa in tension [10]. We attempted to address the possibility of testing the cartilages both in compression and tension, but given the small nature of the cartilages we found compression to be more representative and reliable. The considerable range in the elastic modulus between the studies illustrates the methods for testing the nasal cartilages is not standardised.

The nasal septum was observed to be the major support element in this study, which is consistent with previous studies [2]. The septum is likely to act as the central support structure to the rest of the nasal framework. In comparison with the study by Westreich et al., the alar cartilages were weaker than the septal cartilages in compression, which may be due to the mobile natures of the structures [2]. In this study, we observed that the posterior septal cartilage to be stiffer than the anterior septum. This difference may be accounted for its close placement to the nasal bones. The compressive stiffness of human bone is considerably higher than human cartilage. To prevent mechanical modulus mismatch between the bony tissues and the posterior septum the posterior septum would have a higher elastic modulus than the anterior septum. The alar cartilages were found to be the stiffest compared to the lateral and septal cartilages, when accounting for the anatomical structure of the alar cartilages. As the alar cartilages forms arch structures this may enable the cartilages be take more load in compression and assist in keeping the nasal apertures open [12]. When analysing the relaxation behaviour of the nasal cartilages, the alar cartilage demonstrated a significantly higher stress relaxation and final absolute relaxation stress modulus (*p* < 0.001). This relaxation behaviour could also be accounted for by the alar cartilages requiring to assist in the maintenance of the opening of the nasal apertures.

In addition, to creating a biomechanical map, a histological map of the human nasal cartilages was created. All three nasal cartilages showed characteristics of hyaline cartilage, showing positive staining for collagen and no elastic cartilage staining. Despite the histology of the cartilages being very similar amongst the different cartilages the septal cartilage also showed greater staining for the glycoproteins and proteoglycans as observed by Alcian blue and PAS staining, suggesting a greater glycoprotein and proteoglycan content. Studies have shown that glycoproteins and proteoglycan content contributes to the mechanical properties in hyaline cartilage [13].

Understanding the mechanical properties of the nasal cartilages is hugely significant for tissue engineering fields. Development of a successful implant relies on the structural compatibility of the implant with the surrounding hard and soft tissue. An ideal implant should exhibit similar mechanical properties to the surrounding tissue, as an implant with a higher Young’s Modulus may cause stress shielding, resulting in the failure of the graft [14, 15]. We have therefore provided the range of mechanical properties that the nasal cartilage replacements should be created, taking into account the individual cartilage groups.

Our study has certain limitations. The study evaluated cartilage samples from males with a certain age range. Further study will analyse the cartilage samples taking into consideration ethnicity, gender and age as well as comparison with current materials often used in nasal reconstruction.

## Conclusion

When designing nasal implants, it is important to consider the human nasal framework consists of three cartilages each with different compressive mechanical properties. Furthermore, engineering effective nasal replacements should consider that the human cartilages have specific mechanical properties when accounting for their anatomical structure, with the alar arch demonstrating higher compressive strength.

## Electronic supplementary material

Protocol by which the nasal cartilages were dissected and tested under compression. (A) One point was tested on the lateral cartilages. (B) Four points were tested on the medial and lateral alar cartilage. (C) Sixteen points were tested on the septal cartilage. Four sections were formed from posterior to anterior and four sections were used from top to bottom. Supplementary material 1 (TIFF 1521 kb)

Thickness of the three nasal cartilages groups (mm). P values * < 0.05 ** <0.01 *** p <0.001. Supplementary material 2 (TIFF 1521 kb)

Compression elastic modulus of the nasal cartilages individually. (A) Septal (B) Alar (C) Lateral (D) Grouped. Supplementary material 3 (TIFF 1521 kb)

Compression elastic modulus of the nasal cartilages based on the anatomical structure of the nasal cartilages (MPa). P values * < 0.05 ** <0.01 *** p <0.001. Supplementary material 4 (TIFF 1521 kb)

## References

[1] Oseni A, Crowley C, Lowdell M, Birchall M, Butler PE, Seifalian AM (2012). Advancing nasal reconstructive surgery: the application of tissue engineering technology. J Tissue Eng Regen Med.

[2] Westreich RW, Courtland HW, Nasser P, Jepsen K, Lawson W (2007). Defining nasal cartilage elasticity: biomechanical testing of the tripod theory based on a cantilevered model. Arch Facial Plast Surg.

[3] Al Dayeh AA, Herring SW (2014). Compressive and tensile mechanical properties of the porcine nasal septum. J Biomech.

[4] Colombo V, Cadová M, Gallo LM (2013). Mechanical behavior of bovine nasal cartilage under static and dynamic loading. J Biomech.

[5] Xia Y, Zheng S, Szarko M, Lee J (2012). Anisotropic properties of bovine nasal cartilage. Microsc Res Tech.

[6] Richmon JD, Sage AB, Wong VW, Chen AC, Pan C, Sah RL, Watson D (2005). Tensile biomechanical properties of human nasal septal cartilage. Am J Rhinol.

[7] Richmon JD, Sage A, Wong WV, Chen AC, Sah RL, Watson D (2006). Compressive biomechanical properties of human nasal septal cartilage. Am J Rhinol.

[8] Tavalalok (1989) Proteoglycan & collagen degrading activities of neural proteases from fresh and cryopreserved articular cartilage explants and the chondrocytes. An in vitro biochemical study. University of Calgary. Phd Thesis.

[9] Wood JM, Soldin M, Shaw TJ, Szarko M (2014). The biomechanical and histological sequelae of common skin banking methods. J Biomech.

[10] Alkan Z, Yigit O, Acioglu E, Bekem A, Azizli E, Kocak I, Unal A, Buyuk Y (2011). Tensile characteristics of costal and septal cartilages used as graft materials. Arch Facial Plast Surg.

[11] Bloching MB (2007). Disorders of the nasal valve area. GMS Curr Top Otorhinolaryngol Head Neck Surg.

[12] Lawson M, Trebilcock P (2003). Architectural design in steel.

[13] Zhang L, Hu J, Athanasiou KA (2009). The role of tissue engineering in articular cartilage repair and regeneration. Crit Rev Biomed Eng.

[14] Ramakrishna S, Mayer J, Wintermatel E, Leong KW (2001). Biomedical application for polymer composite materials; a review. Compos Sci Technol.

[15] Yogi Goswami D (2004). The CRC handbook of mechanical engineering.

