# Supplementary figures and images for: Biomechanical characterisation of the human nasal cartilages; implications for tissue engineering
Source: J Mater Sci Mater Med. 2015 Dec 16;27:11. doi: 10.1007/s10856-015-5619-8 (PMC4681753; doi:10.1007/s10856-015-5619-8)

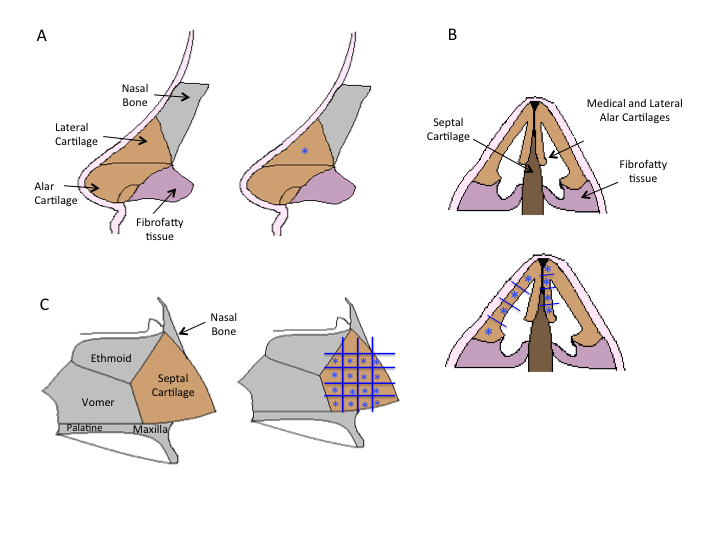

Supplement: Supplementary file 1 — Protocol by which the nasal cartilages were dissected and tested under compression. (A) One point was tested on the lateral cartilages. (B) Four points were tested on the medial and lateral alar cartilage. (C) Sixteen points were tested on the septal cartilage. Four sections were formed from posterior to anterior and four sections were used from top to bottom. Supplementary material 1 (TIFF 1521 kb) [file 10856_2015_5619_MOESM1_ESM.tiff]

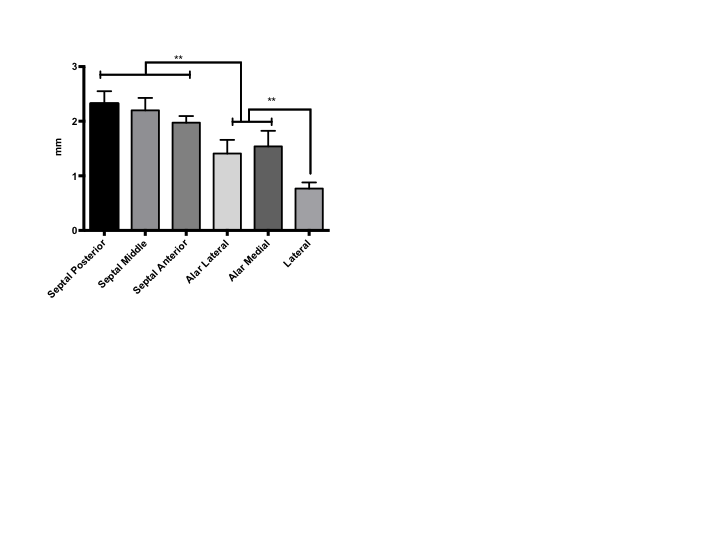

Supplement: Supplementary file 2 — Thickness of the three nasal cartilages groups (mm). P values * < 0.05 ** <0.01 *** p <0.001. Supplementary material 2 (TIFF 1521 kb) [file 10856_2015_5619_MOESM2_ESM.tiff]

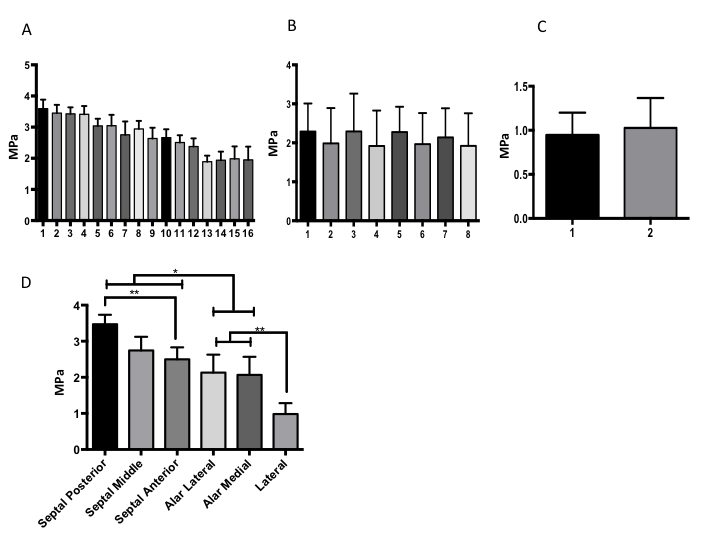

Supplement: Supplementary file 3 — Compression elastic modulus of the nasal cartilages individually. (A) Septal (B) Alar (C) Lateral (D) Grouped. Supplementary material 3 (TIFF 1521 kb) [file 10856_2015_5619_MOESM3_ESM.tiff]

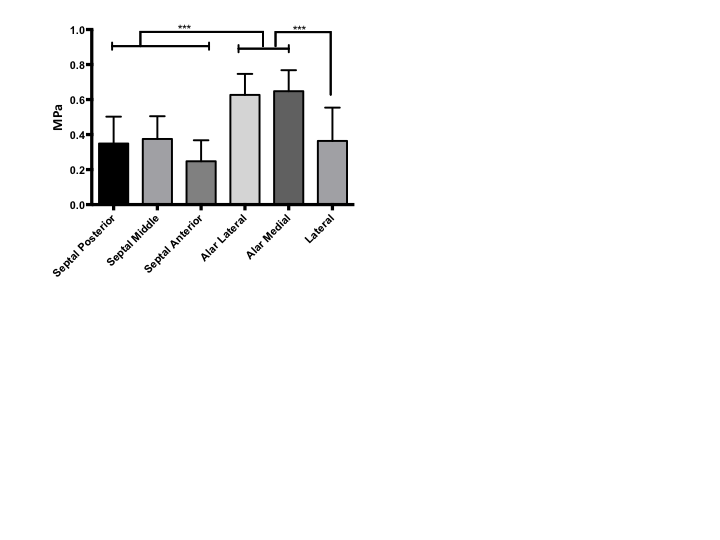

Supplement: Supplementary file 4 — Compression elastic modulus of the nasal cartilages based on the anatomical structure of the nasal cartilages (MPa). P values * < 0.05 ** <0.01 *** p <0.001. Supplementary material 4 (TIFF 1521 kb) [file 10856_2015_5619_MOESM4_ESM.tiff]
